# Hypercalcemia, Anemia, and Acute Kidney Injury: A Rare Presentation of Sarcoidosis

**DOI:** 10.1155/2015/565243

**Published:** 2015-06-23

**Authors:** Neeraj Sharma, Hassan Tariq, Kalpana Uday, Yevgeniy Skaradinskiy, Masooma Niazi, Sridhar Chilimuri

**Affiliations:** ^1^Department of Medicine, Bronx Lebanon Hospital Center, 1650 Selwyn Avenue, Suite No. 10C, Bronx, NY 10457, USA; ^2^Department of Pathology, Bronx Lebanon Hospital Center, 1650 Grand Concourse, Bronx, NY 10457, USA

## Abstract

We discuss a case of a 61-year-old woman who presented with substernal chest pain. She was found to have elevated calcium levels, anemia, and acute kidney injury. The hypercalcemia persisted despite therapy with fluids and bisphosphonates. She was found to have nonparathyroid hormone (PTH) mediated hypercalcemia. The chest X-ray did not reveal any pathology. Our Initial impression was likely underlying hematologic malignancy such as lymphoma or multiple myeloma. A bone marrow biopsy was performed that revealed nonnecrotizing granulomatous inflammation. Further workup revealed elevated vitamin 1,25 dihydroxy level, beta-two microglobulin level, and ACE levels. Noncontrast computed tomography (CT) scan of chest showed bilateral apical bronchiectasis, but did not show any lymphadenopathy or evidence of malignancy. Subsequently, a fiber optic bronchoscopy with transbronchial biopsy showed nonnecrotizing granulomatous inflammation consistent with sarcoidosis. After initiating glucocorticoid therapy, the patient's hypercalcemia improved and her kidney function returned to baseline.

## 1. Introduction

Sarcoidosis is a chronic systemic disease of unknown etiology that is characterized by the formation of immune granulomas in various organs [1]. Although hypercalcemia is a known metabolic complication of sarcoidosis (10–20 percent of patients), it is rarely a presenting manifestation, with clinically significant hypercalcemia occurring in less than 5% of patients [2]. Among the granulomatous disorders, sarcoidosis and tuberculosis are the most common etiologies [3, 4]. Approximately 10–20 percent of patients with sarcoidosis have hypercalcemia [4]. Regardless of the etiology, the complications of hypercalcemia include nephrolithiasis, nephrocalcinosis, nephrogenic diabetes insipidus, renal insufficiency, and polyuria. However, many patients with hypercalcemia and granulomatous disease are asymptomatic. It has been purposed that increased intestinal calcium absorption, induced by high 1,25-dihydroxyvitamin D, is the main abnormality contributing to elevated serum calcium levels. Normally, hypercalcemia suppresses release of PTH and thus calcitriol production; however, in sarcoidosis, activated macrophages produce calcitriol independent of PTH [5, 6]. Furthermore, parathyroid hormone-related protein (PTHrp), which is the usual etiologic agent of humoral hypercalcemia of malignancy, has also been implicated to contribute to hypercalcemia in sarcoidosis [6]. Although sarcoidosis is more prevalent in the African-American population, hypercalcemia is more commonly found in the Caucasian population with sarcoidosis [7]. Here we report a case of a patient who presented with hypercalcemia, anemia, and acute kidney injury who was subsequently diagnosed with sarcoidosis.

## 2. Case Presentation

A 61-year-old woman presented to the emergency department of our hospital with complaint of substernal chest pain for one day. Chest pain was described as being sharp, 7/10 in intensity, being nonradiating, and being with no aggravating or alleviating factors. Chest pain was not associated with any dyspnea, diaphoresis, or palpitations. On review of systems, patient denied any fever, chills, cough, abdominal pain, myalgia, arthralgia, rash, or weight loss.

Her medical comorbidities included heart failure, chronic obstructive pulmonary disease, Parkinson's disease, schizophrenia, hypertension, and diabetes mellitus. She was an exsmoker and did not use any recreational drugs or alcohol. Patient resided in a skilled nursing facility for the past two years and her family history was unknown.

Her physical examination at the time of admission revealed a disoriented and confused elderly woman. She was oriented to only her name. According to nursing home staff the patient at baseline was fully alert and oriented to name, person, and place. Initial vital signs showed temperature 98.8°F (37.1 degrees C), pulse 65 beats per minute, respiratory rate 16 breaths per minute, and blood pressure 119/58 mm of hg, with an oxygen saturation of 100% on room air. She had dry oral mucous membranes and a poor skin turgor. Pupils were equally round and reactive to light and accommodation. There was no jugular venous distention. Chest exam showed bilateral air entry without any adventitious sounds. Cardiovascular exam showed normal heart sounds without murmurs, gallops, or rubs. Abdomen was soft, with no visceromegaly and with normal bowel sounds. Extremities were warm and well perfused without edema, cyanosis, or clubbing.

Laboratory values on admission are shown in Table 1.

The fractional excretion of sodium (Fena) was 1.2%. Anemia workup showed serum iron 36 ug/dL (normal 60–150 ug/dL and SI units: 6.44 umol/L (10.7–26.9)), ferritin 329 ng/mL (normal 15–200 and SI units: 739 pmol/L (33–450)), folate >19.9 ng/mL (normal 3–16 and SI units: 45.09 nmol/L (7–36)), B12 1364 pg/mL (normal 160–950 and SI units: 1006 pmol/L (118–701)), and transferrin saturation of 18%. Workup for hypercalcemia revealed a serum PTH level of 15.8 (normal 10–65 and SI unit: 15.8 ng/L (10–650), total vitamin D 25-OH which was 30.2 ng/mL (normal 14–60 and SI unit 75.38 nmol/L (35–150)), and vitamin D 1,25 dihydroxy level which was elevated at 128 pg/mL (normal 25–45 and SI units 78.5 pmol/L (60–108)). Other pertinent laboratory values are shown in Table 2. Chest X-ray did not show any pathology. Electrocardiogram showed normal sinus rhythm with a 1st degree AV block and an old incomplete LBBB.

**Figure 1:**
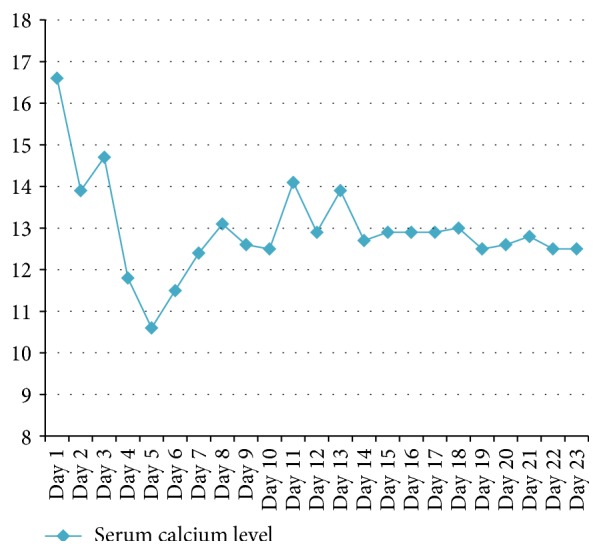
Serum calcium levels during hospitalization.

The patient was initially managed in the medical intensive care unit with intravenous fluids and pamidronate. The serum calcium slowly decreased from 16.6 mg/dL (4.15 mmol/L) to 10.6 mg/dL (2.65 mmol/L).  Figure 1 shows the serum calcium trend during the hospitalization. The renal function improved from serum creatinine of 3.8 mg/dL (335 umol/L) to 2.5 mg/dL (221 umol/L).

**Figure 2:**
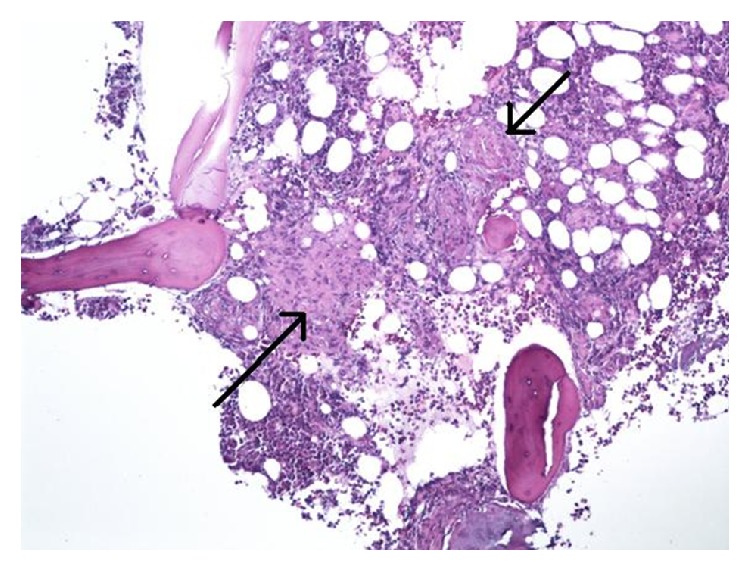
Bone marrow on low power (×200) shows multiple compact nonnecrotizing granulomas (black arrows). The surrounding tissue is comprised of hematopoietic cells with trilineage maturation and bony trabeculae (Hematoxylin and Eosin stain).

**Figure 3:**
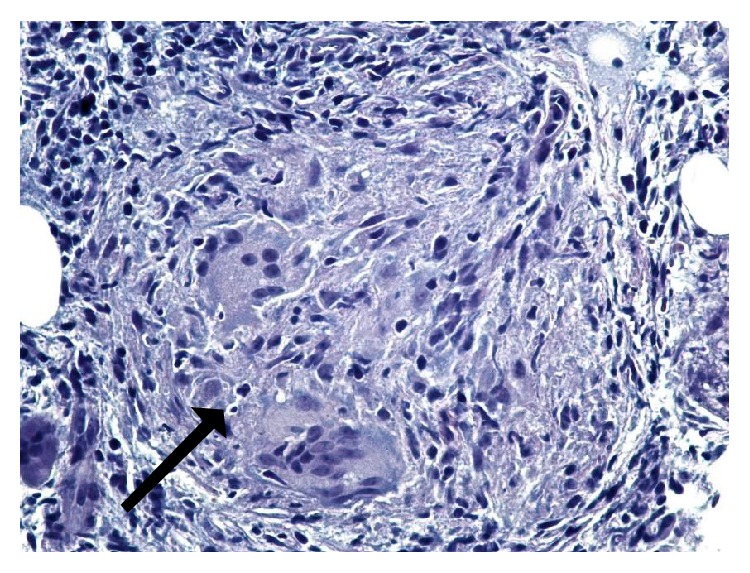
Bone marrow on high power (×400) with well-formed nonnecrotizing granuloma (black arrow) comprised of epithelioid cells, multinucleated giant cells, scant lymphocytes, and no necrosis (Hematoxylin and Eosin stain).

PTH-related peptide was elevated at 45 pg/mL. It was possible that this might have been a false positive because at the time of collection patient had GFR of 12 and serum creatinine of 3.9 mg/dL and PTH-related peptide can be elevated in renal disease. Considering the elevated PTH-related peptide levels, a CT scan of the chest, abdomen, and pelvis without contrast was done which showed upper lobe bronchiectasis and a hypodense 4 cm lesion in left kidney. However, there was no evidence of any malignancy, lymphadenopathy, or splenomegaly. Our initial impression was likely multiple myeloma for which protein electrophoresis was done which revealed a normal serum protein electrophoresis and a urine protein electrophoresis that showed a restriction band. A bone marrow biopsy was performed which revealed nonnecrotizing granulomas (Figures 2 and 3). Additional lab tests showed elevated serum ACE level (113 U/L) and beta-2-microglobulin (14.3 mg/L). Sputum for acid fast bacilli AFBs was sent and tuberculosis (TB) was ruled out.

**Figure 4:**
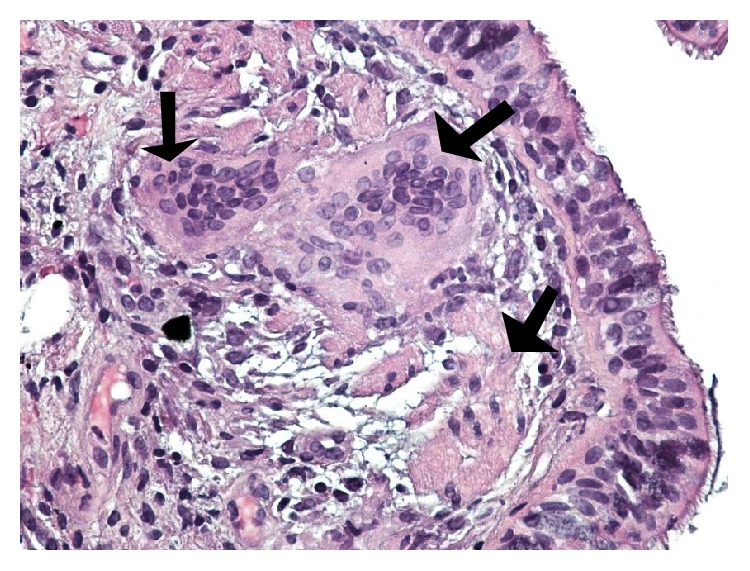
Lung peribronchial tissue on high power (×400) showing nonnecrotizing granuloma comprised mainly of multinucleated giant cells (black arrows) and epithelioid cells. The background shows ciliated pseudostratified columnar epithelium (Hematoxylin and Eosin stain).

Patient underwent Fiberoptic bronchoscopy with bronchoalveolar lavage and transbronchial and endobronchial biopsies. Transbronchial biopsy showed nonnecrotizing granulomatous inflammation consistent with sarcoidosis (Figure 4). The patient was started on prednisone. After receiving therapy with prednisone and hydration the calcium levels decreased and remained stable between 12 mg/dL (3.0 mmol/L) and 12.5 mg/dL (3.13 mmol/L). Renal function and anemia level returned to baseline after receiving starting prednisone and the patient was subsequently discharged to skilled nursing facility on prednisone.

## 3. Discussion

Sarcoidosis was first described in 1899, when Boeck first coined the term “sarcoid” after his findings of “epithelioid cells with large pale nuclei and also a few giant cells” on a skin biopsy [8]. Since then it has remained an enigmatic multisystem granulomatous disease with significant morbidity. Sarcoidosis can affect people of all racial and ethnic backgrounds and usually develops between 20 and 39 years of age [9]. The annual incidence varies throughout the world due to differences in environmental exposures, predisposing HLA alleles, and other genetic factors. The annual incidence among African Americans is three times that among white Americans [9]. Sarcoidosis has an increase in mortality and morbidity among African Americans [9, 10].

The pathogenesis of sarcoidosis involves an inflammatory response that leads to granuloma formation. This inflammatory response involves an interaction between an antigen, human leukocyte antigen (HLA) class II molecules, and T-cell receptors. Once an antigen(s) enters the host, it is phagocytosed by an antigen presenting cell (APC) which processes the antigen and presents it to T-cell receptors on naïve T cells of CD4+ class. This reaction causes a polarization of T cells into a Th1 phenotype followed by proliferation and differentiation leading to formation of a granuloma. According to this process, a combination of any of the three facets, involving the interplay between antigen, HLA molecules, and T cell receptors can initiate development of granulomatous disease. Therefore, there has been interest in investigating either HLA genes, exposure to certain exogenous antigens, or T-cell immune response [11, 12].

Sarcoidosis typically presents with pulmonary manifestations such as bilateral hilar adenopathy (50 percent of cases), with pulmonary reticular opacities, or with skin, joint, or eye lesions. In up to 50 percent of patients, the disease is detected incidentally by radiographic abnormality. Bilateral hilar adenopathy is the most common thoracic manifestation, but 3 to 5 percent of patients with sarcoidosis can have unilateral hilar adenopathy. Pulmonary function tests often reveal restrictive ventilatory defect with a decreased diffusion capacity [13]. Sarcoidosis can involve any organ but in more than 90 percent of patients, it is manifested with pulmonary involvement. Respiratory symptoms include cough, shortness of breath, and chest discomfort. Our patient's only respiratory complaint was vague chest pain. Chest radiographs can be classified into four stages, which represent radiographic patterns and not disease chronicity. Stage 1 is bilateral hilar adenopathy without infiltration. Stage 2 is bilateral hilar adenopathy with infiltration. Stage 3 is infiltration alone. Stage 4 is fibrotic bands, bullae, hilar retraction, bronchiectasis, and diaphragmatic tenting [14]. Our patient interestingly did not have any respiratory complaints and chest radiograph did not reveal any abnormalities. Pulmonary hypertension is another well-known respiratory complication of sarcoidosis. It has been shown that the pulmonary artery pressure is high in 6 to 23 percent of patients while at rest and up to 43 percent of patients with exertion [15]. Fibrosis of the pulmonary vessels is likely the most common mechanism for pulmonary hypertension in patients with sarcoidosis. Our patient was found to have an elevated pulmonary artery pressure with an estimated pulmonary artery systolic pressure of 63 mmHg on transthoracic echocardiograph.

While pulmonary manifestations is the most common presentation of the disease, 30 percent of patients can present with extrapulmonary manifestations involving the skin, eyes, lymph nodes, heart, kidney, and central nervous system. Our patient did not present with any pulmonary nor extrapulmonary manifestations of sarcoidosis, except for acute kidney injury. Even though our patient did not have any skin manifestations, a skin examination is important because biopsy of a cutaneous sarcoid lesion has been shown to have high diagnostic yield. Erythema nodosum signifies a good prognosis during an acute presentation. Other systemic symptoms such as fatigue, night sweat, and weight loss are also common. Lofgren's syndrome, an acute presentation that consists of erythema nodosum, arthritis, and bilateral hilar adenopathy, occurs in 9 to 34 percent of patients with sarcoidosis [16]. Cardiac sarcoidosis is found at autopsy in nearly 25 percent of patients in the United States and up to 50 percent of patients in Japan [17]. Cardiac magnetic resonance imaging is highly sensitive test for cardiac sarcoidosis and can detect minute amounts of sarcoid-related cardiac damage [18]. Neurosarcoidosis is found at autopsy in up to 25% of patients; however neurological disease is only the manifestation in 10 to 17 percent of patients with sarcoidosis [19]. Common symptoms of neurosarcoidosis include headache, ataxia, seizures, and cognitive dysfunction [20].

The diagnosis of sarcoidosis is best supported by a tissue biopsy specimen that reveals noncaseating epithelioid granulomas in a patient with clinical and radiologic findings of the disease [21]. In a patient who presents with Lofgren's syndrome, a diagnosis of sarcoidosis is reasonably certain without biopsy. In all other cases, a biopsy specimen from the organ that is most easily accessible should be obtained such as the skin or lymph nodes. Fiber optic bronchoscopy with transbronchial biopsy has a diagnostic yield of 85 percent [13, 21]. Other initial assessments in the clinic evaluation of sarcoidosis should include pulmonary function tests, ophthalmologic evaluation, and complete blood count and measurement of electrolytes, including measurement of serum angiotensin-converting enzyme (useful to monitor patient compliance).

Skin involvement is also very common, occurring in 25 to 35 percent of patients with sarcoidosis. Lesions such as macules, papules, and plaques can be seen and usually involved the neck, upper back, trunk, and extremities. A disfiguring sarcoid-related skin lesion is Lupus pernio, which is seen as indurated, violaceous lesions on the face. Lupus pernio is more common in woman and is associated with chronic sarcoidosis [22].

An uncommon extrapulmonary involvement of sarcoidosis is hypercalcemia and renal disease. It has been shown that 10–20 percent of patient with sarcoidosis have hypercalcemia [23]. And 10 percent have renal calculi. These abnormalities of calcium metabolism are due to dysregulated production of 1,25-dihydroxyvitamin D by activated macrophages trapped in pulmonary alveoli and granulomatous inflammation [5, 6, 24]. 1,25-dihydroxyvitamin D increases serum calcium by mainly increasing intestinal calcium absorption but also acts on the bone and kidney to increase serum calcium as well [24]. In the 1970s, it was shown that 1,25-dihydroxyvitamin D levels were elevated in hypercalcemic patients with sarcoidosis and, later, Adams and colleagues showed that the increased 1,25-dihydroxyvitamin D levels were due to production by the pulmonary alveolar macrophages [24].

Hypercalcemia is most often caused by primary hyperparathyroidism or malignancy. However there are many other conditions to consider, such as hypocalciuric hypercalcemia, multiple myeloma, vitamin A and vitamin D intoxication, thyrotoxicosis, tuberculosis, fungal infections, thyrotoxicosis, lymphoma, and sarcoidosis. Causes can be divided into PTH-mediated (high or normal PTH levels) and non-PTH mediated. In PTH mediated hypercalcemia the two differential diagnoses to consider are primary hyperparathyroidism and familial hypocalciuric hypercalcemia. These entities are differentiated bases upon urinary calcium level. Elevated urinary calcium in a patient with PTH mediated hypercalcemia signifies primary hyperparathyroidism. The differential is much broader in non-PTH mediated hypercalcemia and requires measurement of PTH-related peptide levels, 1,25 vitamin D, vitamin 25 OH. An elevated PTH-related peptide in non-PTH mediated hypercalcemia indicates malignancy as the cause of hypercalcemia. An elevated 1,25 vitamin D level in non-PTH mediated hypercalcemia indicates lymphoma or granulomatous disease. If PTH-related peptide and 1,25 vitamin D levels are normal, then next step requires measurement of vitamin 25 OH, SPEP, UPEP, TSH, and vitamin A levels. Other miscellaneous causes of hypercalcemia to consider are chronic lithium therapy, thiazide diuretics, adrenal insufficiency, and rhabdomyolysis [25, 26].

The incidence and prevalence of renal involvement in sarcoidosis remains unclear [27]. It has been suggested that renal involvement occurs in 35 to 50 percent of patients of sarcoidosis [27, 28]. Primary renal manifestations are nephrolithiasis, nephrocalcinosis, and acute interstitial nephritis with or without granuloma formation. The nephrocalcinosis and nephrolithiasis are due to hypercalcemia resulting from increased GI absorption of calcium due to increased 1,25-dihydroxyvitamin D levels by activated mononuclear cells. Nephrolithiasis and nephrocalcinosis may be initial presenting feature of sarcoidosis [29]. However, only 2 to 3% of patients with sarcoidosis will present with nephrolithiasis as the first manifestation [29]. The stones are typically composed of calcium oxalate. Patients with nephrocalcinosis may present with elevated creatinine and a benign urinalysis. These patients may have polyuria, which may be due to central diabetes insipidus from granulomatous infiltration of the hypothalamus [30]. It has been shown that glucocorticoids can improve calcium metabolism in patients with sarcoidosis. The other renal lesion associated with sarcoidosis is interstitial nephritis, which occurs in 20 percent of patients with sarcoidosis [31]. However, sarcoid-related interstitial nephritis is typically seen during the initial presentation of sarcoidosis and rarely in patients with long-standing sarcoidosis [32]. The urinary manifestations of sarcoid nephritis are nonspecific as the urinalysis is typical of chronic tubulointerstitial diseases [33]. The diagnosis of sarcoid nephritis is suggested by a renal biopsy which reveals normal glomeruli and interstitial infiltration with mononuclear cells and noncaseating granulomas in the interstitium [32, 33]. The findings are suggestive, but not diagnostic, of sarcoidosis. Sarcoid nephritis is a disease of exclusion; therefore other etiologies of interstitial nephritis must be ruled out and there must be extrarenal manifestations of granulomatous disease to be confident about diagnosis of sarcoid nephritis. Patients with sarcoidosis may occasionally also present with glomerular disease; however the relationship to sarcoidosis has not been proven. In all the types of renal lesions caused by sarcoidosis, glucocorticoids have been shown to improve renal function [32].

The cornerstone of treatment of hypercalcemia due to sarcoidosis is glucocorticoids. Prednisone, 15–25 mg/day, is the drug of choice to reduce the overproduction of 1,25-dihydroxyvitamin D levels. In addition, reducing calcium intake (to under 400 mg/day), reducing oxalate intake, and avoiding sun exposure are also recommended in treatment of hypercalcemia in granulomatous disease. Chloroquine is one alternative therapy that may also be considered [34]. Although the mechanism in unclear, it is shown to impair granuloma formation. However retinal toxicity is the major concern with the use of chloroquine, although hydroxychloroquine carries less risk of retinopathy. Ketoconazole is another alternative to glucocorticoids [35].

This was a rare case of a patient with an unusual presentation of sarcoidosis with hypercalcemia, anemia, and acute kidney injury being the initial presentation. Hypercalcemia is rarely a presenting manifestation, with clinically significant hypercalcemia occurring in less than 5% of patients. Awareness about variegated presentation of sarcoidosis will prompt early institution of targeted therapy and prevent long-term sequelae of this potentially fatal condition.

## Figures and Tables

**Table 1:** Laboratory values on admission (reference range in parenthesis).

Laboratory test	Results on admission	SI units
White blood cell count	8.5 k/uL (4.8–10.8)	8.5 × 10^9^/L (4.5–11.0)
Hemoglobin	7.9 g/dL (12.0–16.0)	79 g/L (140–175)
Hematocrit	23.9% (42%–51%)	0.24 proportion of 1 (0.41–0.50)
Platelets	233 k/uL (150–400)	233 × 10^9^/L (150–350)
Sodium	138 mEq/L (135–145)	138 mmol/L (136–142)
Potassium	4.1 mEq/L (3.5–5.0)	4.1 mmol/L (3.5–5.0)
Chloride	94 mEq/L (98–108)	94 mmol/L (96–106)
Bicarbonate	29 mEq/L (24–30)	29 mmol/L (21–28)
Blood urea nitrogen	56 mg/dL (8–26)	19.9 mmol/L (2.9–8.2)
Creatinine	3.8 mg/dL (0.5–1.5)	335 umol/L (53–106)
Calcium	16.6 mg/dL (8.5–10.5)	4.15 mmol/L (2.05–2.55)
Ionized calcium	1.93 mmol/L (1.15–1.27)	1.93 mmol/L (1.15–1.27)
Magnesium	4.6 mg/dL (1.5–2.7)	2.30 mmol/L (0.65–1.05)
Total protein	7.1 g/dL (5.8–8.3)	71 g/L (60–80)
Albumin	4.1 g/dL (3.2–4.6)	40 g/L (35–50)
AST	15 U/L (9–36)	0.25 ukat/L (0.17–0.51)
ALT	5 U/L (5–40)	0.08 ukat/L (0.17–0.68)
Alkaline phosphatase	142 U/L (43–160)	2.37 ukat/L (0.5–2.0)
Total bilirubin	0.2 mg/dL (0.2–1.1)	3.42 umol/L (5.0–21)
Direct bilirubin	0.1 mg/dL (0.0–0.3)	1.71 umol/L (1.7–5.1)

**Table 2:** Pertinent laboratory values during hospitalization (SI units are in parenthesis).

Laboratory test	Admission	Day 5	Day 10	Day 15	Day 20	Day 25
Serum calcium	16.6 (4.15 mmol/L)	10.6 (2.65 mmol/L)	12.5 (3.13 mmol/L)	12.9 (3.23 mmol/L)	12.6 (3.15 mmol/L)	12.5 (3.13 mmol/L)
Serum phosphorus	6.3 (2.03 mmol/L)	2.8 (0.90 mmol/L)	3.4 (1.10 mmol/L)	3.4 (1.10 mmol/L)	4.3 (1.39 mmol/L)	5.0 (1.62 mmol/L)
Serum magnesium	4.6 (2.30 mmol/L)	2.3 (1.15 mmol/L)	1.6 (0.80 mmol/L)	3.4 (1.70 mmol/L)	1.8 (0.9 mmol/L)	2.0 (1.00 mmol/L)
BUN	56 (19.9 mmol/L)	30 (10.7 mmol/L)	25 (8.9 mmol/L)	30 (10.7 mmol/L)	32 (11.4 mmol/L)	54 (19.2 mmol/L)
Creatinine	3.8 (335 umol/L)	2.8 (247 umol/L)	2.4 (212 umol/L)	2.3 (203 umol/L)	1.9 (167 umol/L)	2.1 (185 umol/L)
